# Bioconversion of non-food corn biomass to polyol esters of fatty acid and single-cell oils

**DOI:** 10.1186/s13068-023-02260-z

**Published:** 2023-01-17

**Authors:** Guang-Lei Liu, Xian-Ying Bu, Chaoyang Chen, Chunxiang Fu, Zhe Chi, Akihiko Kosugi, Qiu Cui, Zhen-Ming Chi, Ya-Jun Liu

**Affiliations:** 1 College of Marine Life Sciences, Ocean University of China, Qingdao, 266101 People’s Republic of China; 2 Laboratory for Marine Biology and Biotechnology, Pilot National Laboratory for Marine Science and Technology, Qingdao, 266101 China; 3 CAS Key Laboratory of Biofuels, Shandong Provincial Key Laboratory of Synthetic Biology, Qingdao Institute of Bioenergy and Bioprocess Technology, Chinese Academy of Sciences, Qingdao, China; 4 Shandong Energy Institute, Qingdao, China; 5 Qingdao New Energy Shandong Laboratory, Qingdao, China; 6 Dalian National Laboratory for Clean Energy, Qingdao, China; 7 University of Chinese Academy of Sciences, Beijing, China; 8 Biological Resources and Post-Harvest Division, Japan International Research Center for Agricultural Sciences (JIRCAS), 1-1 Ohwashi, Tsukuba, Ibaraki Japan

**Keywords:** Lignocellulose, Glycolipids, Polyol esters of fatty acids, Saccharification, Yeast fermentation

## Abstract

**Background:**

Lignocellulose is a valuable carbon source for the production of biofuels and biochemicals, thus having the potential to substitute fossil resources. Consolidated bio-saccharification (CBS) is a whole-cell-based catalytic technology previously developed to produce fermentable sugars from lignocellulosic agricultural wastes. The deep-sea yeast strain *Rhodotorula* *paludigena* P4R5 can produce extracellular polyol esters of fatty acids (PEFA) and intracellular single-cell oils (SCO) simultaneously. Therefore, the integration of CBS and P4R5 fermentation processes would achieve high-value-added conversion of lignocellulosic biomass.

**Results:**

The strain P4R5 could co-utilize glucose and xylose, the main monosaccharides from lignocellulose, and also use fructose and arabinose for PEFA and SCO production at high levels. By regulating the sugar metabolism pathways for different monosaccharides, the strain could produce PEFA with a single type of polyol head. The potential use of PEFA as functional micelles was also determined. Most importantly, when sugar-rich CBS hydrolysates derived from corn stover or corncob residues were used to replace grain-derived pure sugars for P4R5 fermentation, similar PEFA and SCO productions were obtained, indicating the robust conversion of non-food corn plant wastes to high-value-added glycolipids and lipids. Since the produced PEFA could be easily collected from the culture via short-time standing, we further developed a semi-continuous process for PEFA production from corncob residue-derived CBS hydrolysate, and the PEFA titer and productivity were enhanced up to 41.1 g/L and 8.22 g/L/day, respectively.

**Conclusions:**

Here, we integrated the CBS process and the P4R5 fermentation for the robust production of high-value-added PEFA and SCO from non-food corn plant wastes. Therefore, this study suggests a feasible way for lignocellulosic agro-waste utilization and the potential application of P4R5 in industrial PEFA production.

**Supplementary Information:**

The online version contains supplementary material available at 10.1186/s13068-023-02260-z.

## Background

Lignocellulosic biomass (LCB) is known as an abundant, widespread, and recalcitrant substrate containing cellulose, hemicellulose, and lignin as the main components [1]. LCB is difficult to degrade and utilize, although it has unique material properties as carbon feedstock compared with other renewable resources. With the urgent global requirement for carbon neutrality, effective LCB application is of particular importance [2].

In the last decades, integrated strategies, including pretreatment, saccharification, and sequential fermentation procedures in principle, have been developed for the utilization of various LCB materials [3, 4]. The LCB conversion strategies are mainly known as simultaneous saccharification and fermentation (SSF) and consolidated bioprocessing (CBP), employing cellulases from fungi or cellulosome-producing strains as the biocatalysts to produce target products via separated or one-pot reaction, respectively [5]. Consolidated bio-saccharification (CBS) is a recently developed technology based on CBP, aiming at the efficient conversion of lignocellulose to fermentable sugars using engineered *Clostridium* *thermocellum* as the whole-cell biocatalyst [6, 7].

In terms of industrial application, the techno-economic feasibility of the LCB bioconversion technologies should be evaluated based on key factors including biomass substrate, process cost, and potential products [8]. Compared to off-site saccharification using independently produced fungal cellulases, the CBS strategy helps to reduce costs because the alive strains can continuously produce the extracellular cellulosome, a type of multi-enzyme complex that is capable of efficient cellulose degradation [9–11]. Furthermore, the sugar-rich CBS hydrolysates can be subsequently used to produce high-value-added products. For example, a newly isolated fungus that is capable of co-utilizing glucose and xylose has been previously used to produce pullulan with wheat straw-derived hydrolysate as the nutrient [12].

Given the complex composition of LCB hydrolysates and the required carbon conversion efficiency in terms of industrial production [13], it is critical to explore novel fermenting strains that can tolerate potential inhibitory compounds derived from LCB substrates and co-utilize glucose and xylose [5]. Yeast species of the genus *Rhodotorula* are non-conventional, pink-colored, and oleaginous basidiomycetous fungi, and are famous for their excellent lipid and carotenoid accumulation ability [14–16]. Furthermore, *Rhodotorula* species can effectively metabolize xylose and have excellent tolerance toward representative inhibitors from LCB biomass hydrolysates [17, 18]. Therefore, *Rhodotorula* strains are considered the ideal candidates to produce high-value-added products using sugar-rich LCB hydrolysates as inexpensive nutrients [19].

Glycolipids are determined as the most promising biosurfactants because of their excellent surface activities and versatile interfacial and biochemical properties [20]. Limited species of *Rhodotorula*, including *R.* *babjevae*, *R.* *taiwanensis*, *R.* *graminis*, and *R.* *paludigena* have been reported to synthesize and secrete a new type of glycolipid, termed polyol esters of fatty acids (PEFA) [21–25]. PEFA is composed of both a polyol head group (either d-mannitol or d-arabitol) with varying degrees of acetylation and an acetylated (*R*)-3-hydroxy fatty acid (primarily C16:0 or C18:0) [23] and shows good emulsification, low critical micelle concentration (CMC), and efficient antifungal activity. Thus, PEFA is considered a type of high-grade glycolipid for chemical and medical applications [22, 24].

**Fig. 1:**
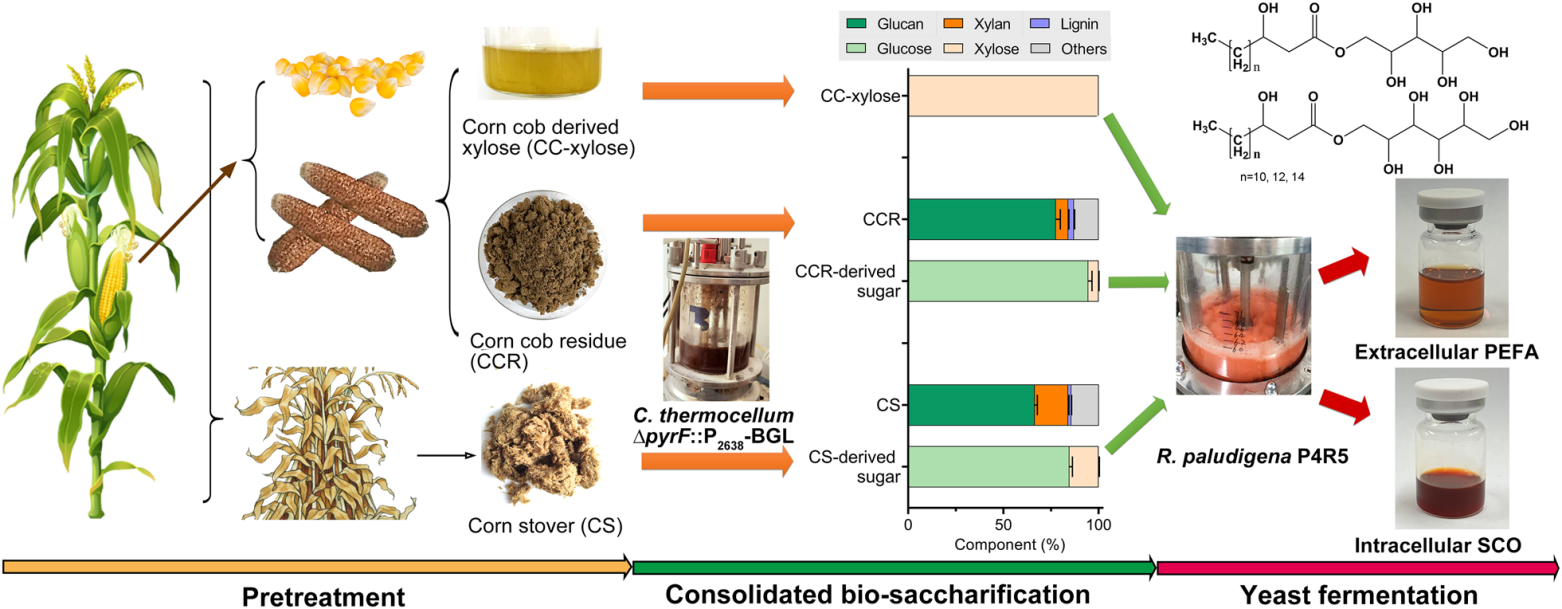
Schematic representation of the whole process for PEFA and SCO production from non-food corn biomass by coupling consolidated bio-saccharification with yeast fermentation of P4R5

So far, all the reported PEFA production processes use limited carbon sources of glucose and sucrose, and the titer ranges from 8.5 to 31.2 g/L [21, 23, 26]. Thus, it remains challenging to develop feasible bioconversion processes for PEFA production from agricultural LCB wastes. It is reported that over 200 million metric tons of non-food biomass from corn plants, mainly corncob and corn stover, can be collected globally, providing abundant LCB substrates [27, 28]. Therefore, with corn plant wastes as the example of lignocellulosic agro-wastes, we would like to develop a reference process for the bioconversion of inexpensive lignocellulosic biomass to high-value-added biochemicals. We previously isolated a yeast strain *R.* *paludigena* P4R5 from deep-sea sediment in the South Pacific. This strain has an excellent ability to produce high yields of PEFA and single-cell oils (SCO) simultaneously [22]. In this study, the strain P4R5 was proven to co-assimilate glucose and xylose to produce PEFA and SCO. Subsequently, we coupled the P4R5 fermentation with the CBS process to convert corncob-derived syrup (mainly xylose), corncob residue, and corn stover to PEFA and SCO (Fig. 1). High production of extracellular PEFA and intracellular SCO by P4R5 was detected despite the different sugar compositions of the hydrolysates. Furthermore, a semi-continuous process was constructed to produce PEFA from LCB hydrolysates requiring no complex product separation. Therefore, we achieved bioconversion from non-food corn plant wastes to high-value-added extracellular PEFA and intracellular lipids. This work provides a good reference for the effective utilization of agricultural LCB wastes and a promising microbial cell factory for PEFA production.

## Results

### *R. paludigena* P4R5 utilized various monosaccharides for PEFA and SCO production

*R.* *paludigena* P4R5 is a deep-sea yeast previously isolated from sediment samples in the South Pacific. With glucose as the carbon source, P4R5 can simultaneously produce intracellular SCO and extracellular PEFA at high levels [22]. LCB-derived polysaccharide consists of cellulose and hemicellulose, which can be hydrolyzed to C6 (glucose) and C5 (mainly xylose) sugars, respectively [29]. To determine whether P4R5 is suitable for fermenting LCB hydrolysates containing both C5 and C6 sugars [5], cell growth and PEFA production of the yeast strain were monitored using glucose and xylose as the carbon sources. To comprehensively understand the monosaccharide metabolism of P4R5, l-arabinose, d-arabinose, and fructose were also tested, given that the corn fiber also has arabinose as one of the main components [30].

**Fig. 2:**
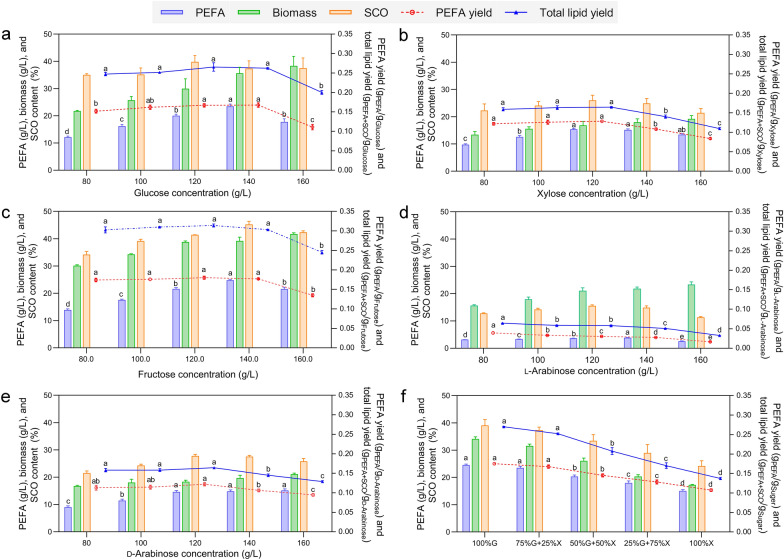
Effects of the different concentrations of monosaccharides on PEFA production. **a**–**e** PEFA production with d-glucose, d-xylose, d-fructose, d-arabinose, and l-arabinose as the sole carbon source. **f** The effect of glucose (G) to xylose (X) ratios on PEFA production. The PEFA titer (g/L), cell dry weight (biomass, g/L), SCO content (%), PEFA yield (g_PEFA_/g_sugar_), and total lipid yield (g_PEFA_ + g_SCO_/g_sugar_) were determined to evaluate the PEFA production. Values were the means of three independent determinations. ^abcd^Data with different superscripts differ (*p* < 0.05)

The results indicated that P4R5 could use all tested monosaccharides as the carbon source. The optimal concentration for glucose and xylose were 140 and 120 g/L, the obtained PEFA titer were 23.5 ± 0.4 and 15.4 ± 0.1 g/L, and the obtained PEFA yields were 0.17 and 0.13 g/g, respectively (Fig. 2a, b). High levels of cell biomass, SCO, and PEFA production of P4R5 were also detected for fructose fermentation. The PEFA titer and yield were as high as 24.8 ± 0.4 g/L and 0.18 g/g, respectively, with 140 g/L of fructose as the carbon source (Fig. 2c). In terms of arabinose, the fermentation of both l-arabinose and d-arabinose was tested, resulting in PEFA titers of 3.87 ± 0.1 g/L and 14.8 ± 0.5 g/L, respectively (Fig. 2d, e). Interestingly, although P4R5 produced less PEFA using l-arabinose than other monosaccharides, with the isomer d-arabinose that is rarely present in nature as the carbon source, the PEFA production was comparable to that with xylose. This result indicated that P4R5 could assimilate the main monosaccharides derived from LCB for cell growth and PEFA production and employed different pathways for l-arabinose and d-arabinose assimilation.

### Robust *R. paludigena* P4R5 fermentation using mixed glucose and xylose

Glucose and xylose are the main monosaccharide components in CBS hydrolysates with varied ratios depending on LCB substrate composition [5]. To investigate whether P4R5 could co-utilize C5 and C6 sugars derived from LCB hydrolysates, P4R5 fermentation was further carried out using mixed glucose and xylose as the carbon source. We also tested the effects of glucose-to-xylose ratios on cell growth, SCO accumulation, and PEFA production of P4R5. As shown in Fig. 2f, the total lipid yield showed a decreasing trend from 0.27 to 0.14 g_PEFA+SCO_/g_sugar_ with varied glucose-to-xylose ratio from 100%:0 to 0:100%, suggesting a preference for glucose as a substrate over xylose. Nevertheless, it is noteworthy that the PEFA titers and yields and SCO content were not significantly influenced when xylose accounted for one-quarter of the total sugar (*p* > 0.05) (Fig. 2f), indicating that P4R5 was capable of robust utilization of mixed sugars containing no more than 25% of xylose. Thus, the coupling of P4R5 fermentation with the LCB saccharification would lead to a complete process of high-value-added LCB utilization.

### PEFA produced from different sugars showed similar fatty acid profiles but different polyol heads

**Fig. 3:**
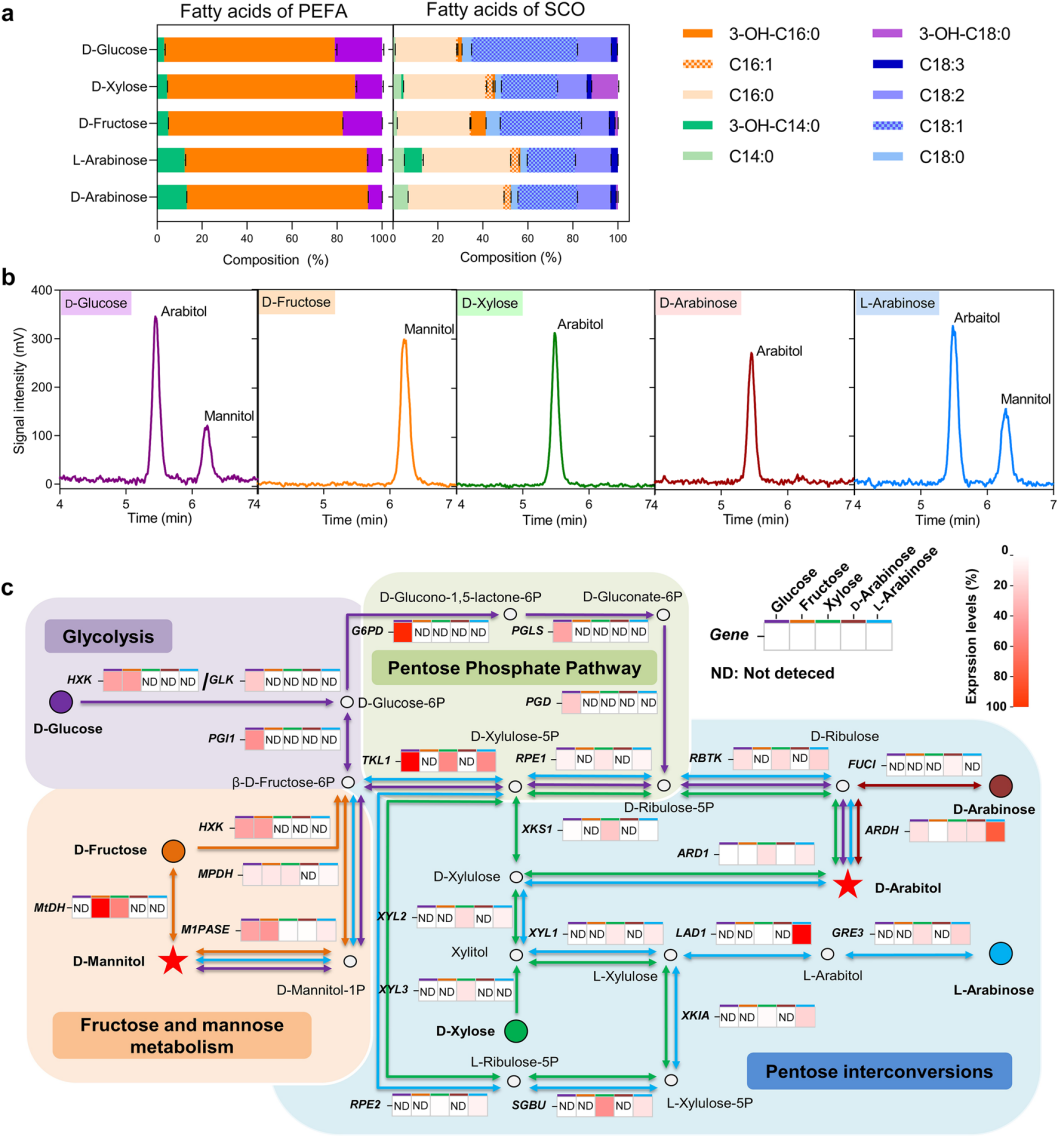
Characterization of PEFA and SCO produced by P4R5 with different carbon sources. **a** The fatty acid compositions of PEFA and SCO derived from different monosaccharides. Three replicates were prepared for calculating average values and standard errors. **b** HPLC analysis of the polyol groups of the produced PEFA from d-glucose, d-xylose, d-fructose, d-arabinose, and l-arabinose. **c** The overall representation of the metabolic pathways of P4R5 for arabitol and mannitol production from glucose, fructose, xylose, d-arabinose, and l-arabinose. Color-filled cycles indicate the monosaccharides. Arrows indicate the relative metabolic pathways of the monosaccharides with the same color. Red stars indicate the products of arabitol and mannitol. The transcriptional levels of the key genes under different carbon resource conditions were labeled with squares with colored top borders. Heat maps showed the gene transcriptional levels relative to that of *β-ACTIN*

As the cell growth and PEFA production of P4R5 varied with different monosaccharides as carbon sources (Fig. 2), we further analyzed the compositions of PEFA and intracellular SCO derived from various sugars. As shown in Fig. 3a, the extracellular PEFA produced from different monosaccharides contained 3-OH fatty acids from C_14_ to C_18_, with 3-OH-C_16:0_ accounting for the vast majority (Fig. 3a). Further investigation of the fatty acid profiles showed that PEFA derived from hexose (glucose and fructose) contained more 3-OH-C_18:0_ but less 3-OH-C_14:0_ and 3-OH-C_16:0_ compared with that derived from pentose (xylose, l-arabinose, and d-arabinose). It is known that hydroxy fatty acids have wide applications in chemical, food, cosmetic and medical fields for their polymerizability and various bioactivities, such as antimicrobial, anti-inflammatory, and anticancer [31]. Therefore, together with the capability of co-utilizing C5 and C6 sugars, P4R5 could be used as a promising feedstock for the production of hydroxy fatty acids from non-food LCB hydrolysate. In terms of the intracellular SCO, C_16_ fatty acids (C_16:0_ and C_16:1_) and C_18_ fatty acids (C_18:0_, C_18:1_, and C_18:2_) were detected as the major components under all tested fermentation conditions (Fig. 3a). Such SCO fatty acid profile together with the high production enabled P4R5 to be a potential feedstock for high-quality biodiesel production [22, 32].

It is noteworthy that, for SCO produced from hexose, C_16_ and C_18_ fatty acids accounted for 27.8% to 32.9% and 57.4% to 69.0%, respectively, while for SCO produced from pentose, C_16_ and C_18_ fatty acids accounted for 39.7% to 45.6% and 42.9% to 46.7%, respectively (Fig. 3a). Thus, hexose-derived SCO contained significantly more C_18_ but less C_16_ fatty acids compared with pentose-derived SCO (*p* < 0.01). Together with the similar pattern of PEFA fatty acids, this result indicated that the fatty acid elongation in P4R5 might be preferred under the growth condition with hexose as the carbon resource compared with pentose. In addition, because 3-hydroxyl fatty acids were also detected in SCO produced from different monosaccharides, the hydroxy fatty acids could be synthesized intracellularly.

Although PEFA derived from various sugars showed similar fatty acid profiles, we speculated on the substrate specificity of PEFA production on the polyol heads. As indicated in Fig. 3b, the strain that grew on glucose or l-arabinose produced a mixture of arabitol- and mannitol-headed PEFA, and the arabitol-headed PEFA accounted for the predominant component. Such results were predictable because all previously reported PEFA-producing strains produce mixed PEFA molecules with different polyol head groups [26, 33]. Interestingly, rather than producing mixed PEFA, P4R5 only produced arabitol-type PEFA with xylose or d-arabinose as the carbon source, and the fructose-grown cells only produced mannitol-type PEFA. This result confirmed that P4R5 was capable of producing pure PEFA with only one type of polyol-head group by simply adjusting the carbon sources.

The glucose-derived PEFA produced by P4R5 has been confirmed to be a promising biosurfactant [22]. The amphiphilicity of PEFA also enables its potential application in preparing self-assembling functional micelles [20]. Thus, we prepared micelles using PEFA produced from various sugars through one-step self-assembly. All PEFA micelles showed similar particle size, PDI, and zeta potential, ranging from 128.00 to 151.23 nm, 0.21 to 0.27, and − 22.70 to 28.73 mV, respectively (Table 1). In addition, PEFA derived from various sugars showed relatively consistent CMC values of 7.64–7.82 mg/L (Table 1), which were lower than those of previously reported rhamnolipids (50–200 mg/L) and sophorolipids (9.5 mg/L) [34]. These results confirmed that P4R5 could produce high-grade PEFA robustly using various sugars, and the produced PEFA could be used to prepare nano-micelles with uniform particle size distribution.

**Table 1:** Characterization of nano-micelles prepared using different PEFA produced by P4R5 grown on different carbon sources

Carbon source	PEFA CMC (mg/L)	Nano-micelles properties		
		Particle size (nm)	PDI	Zeta potential (mV)
Glucose	7.69	128.93 ± 0.65	0.37 ± 0.01	− 28.07 ± 0.47
Xylose	7.74	151.23 ± 2.79	0.23 ± 0.00	− 23.73 ± 0.40
Fructose	7.82	143.17 ± 1.63	0.23 ± 0.01	− 28.73 ± 0.70
d-Arabinose	7.71	128.00 ± 4.31	0.21 ± 0.01	− 26.13 ± 0.51
l-Arabinose	7.64	137.73 ± 2.63	0.21 ± 0.01	− 22.70 ± 0.46

### The substrate specificity of PEFA production depends on the sugar metabolism regulation

To further understand the substrate-coupled PEFA production in terms of polyol head, we analyzed the sugar metabolism pathways for arabitol and mannitol synthesis of P4R5 by analyzing the transcription of key genes based on the genomic data (Additional file 1: Tables S1, S2).

With glucose or l-arabinose as the carbon source, we detected the expression of *M1PASE* and *MPHD* genes for mannitol synthesis. The transcription of genes involved in the pentose phosphate pathway and pentose interconversion pathway for arabitol synthesis was also determined (Fig. 3c). This result explained the presence of both arabitol- and mannitol-head in produced PEFA (Fig. 3b). In comparison, when using xylose or d-arabinose as the carbon source, no expression of *M1PASE* and *MPHD* was observed, which was also consistent with the result shown in Fig. 3b that only arabitol was detected. With fructose as the carbon source, *MtDH*, the key gene for mannitol synthesis from fructose, showed a relatively high expression level. However, the expression of *ARHD* and *ARD1* involved in arabitol synthesis was not detected, which could explain why only mannitol was present in fructose-derived PEFA. These results indicated that P4R5 could regulate the arabitol and mannitol synthesis at the transcriptional level based on the utilized carbon source, and accordingly influence the type of polyol head of produced PEFA.

### Bioconversion of non-food corn plant wastes to high-value-added PEFA and SCO

Following the reported CBS process, the strain *C.* *thermocellum* ∆*pyrF*::p2638-BGL was used as the biocatalyst to produce sugar-rich LCB hydrolysates [19]. Alkali-pretreated non-food corn plant wastes, including CS and CCR, were used as the substrates. The composition of pretreated corn biomass was determined to be 66.4% glucan, 17.5% xylan, 1.7% lignin for CS, and 77.6% glucan, 6.5% xylan, 3.0% lignin for CCR (Fig. 1). Through chemical composition determination of pretreated CS and CCR [35], we also detected 2.6% of arabinose in sulfuric acid-hydrolyzed pretreated CS, indicating the presence of arabinoxylan in pretreated CS. But no arabinose was observed in pretreated CCR according to HPLC analysis (Additional file 2: Fig S1). The results indicated that both pretreated CS and CCR contained glucose and xylose as the main monosaccharide components, and pretreated CS contained less glucan but more xylan compared with CCR.

**Fig. 4:**
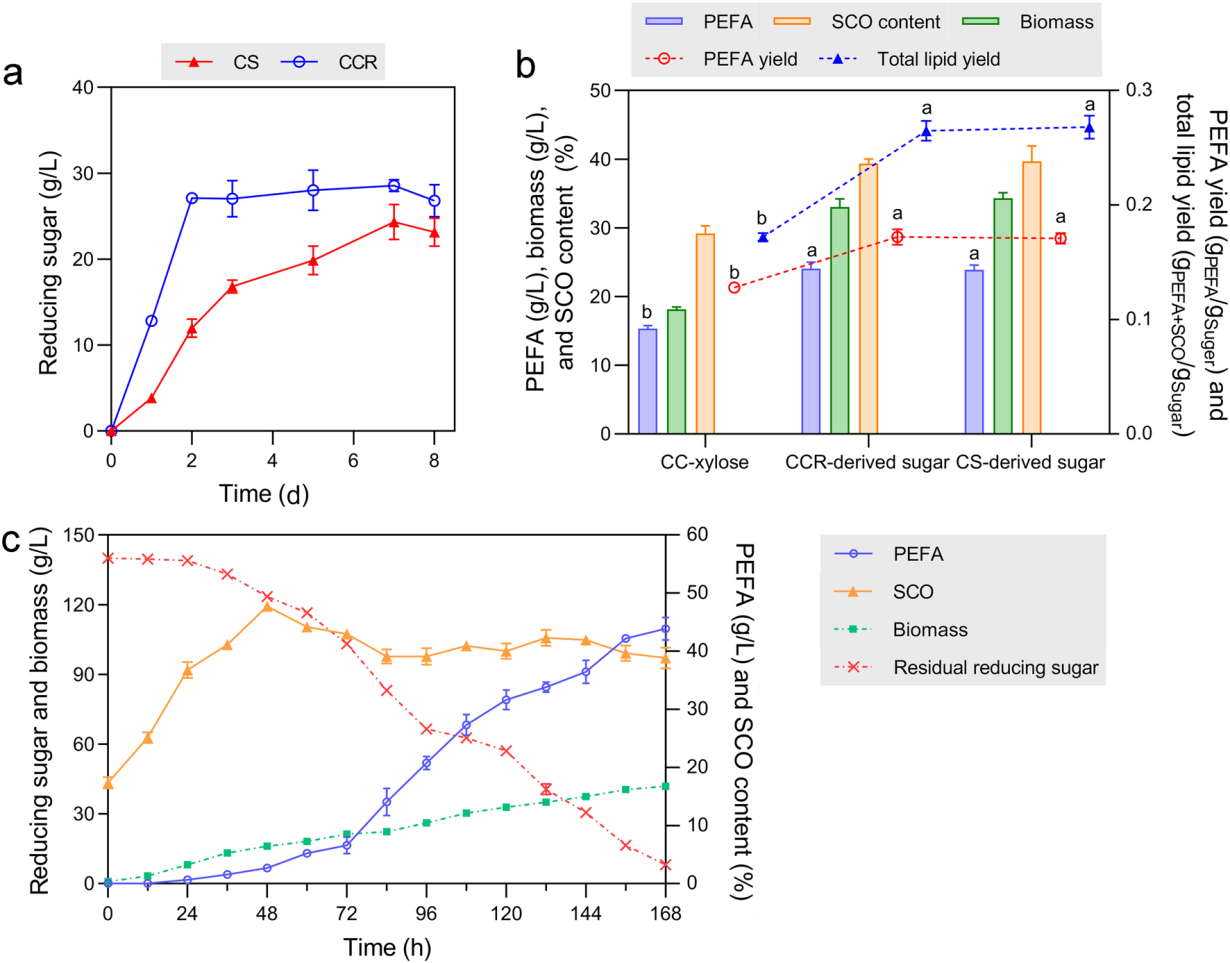
Production of PEFA and SCO from non-food corn biomass. **a** The production of sugar-rich CBS hydrolysates from pretreated CS and CCR. The produced reducing sugar was determined by the DNS method. **b** The production of PEFA and SCO from CS- and CCR-derived sugar and CC-xylose. PEFA titer, biomass, SCO content, PEFA yield, and total lipid yield were determined to investigate the effect of different sugar-rich hydrolysates on lipid production. ^abc^Data with different superscripts differ (*p* < 0.05). **c** 10-L batch fermentation of P4R5 for the production of PEFA and SCO from CCR-derived sugar. The time course of PEFA titer, biomass, SCO content, and residual reducing sugar were determined within 168 h. Three replicates were prepared for statistical analysis

The CBS process of 40 g/L pretreated CS or CCR lasted for 8 days (Fig. 4a). For CCR, the maximal reducing sugar production (28.5 g/L) was achieved in 2 days. For CS, 7 days were required for the complete saccharification to obtain 24.3 g/L of reducing sugar. The glucose proportions in the purified CS- and CCR-derived sugar were 84.7% and 94.6%, respectively (Fig. 1). The glucose-to-xylose ratio in the hydrolysates varied (84.7–15.3% for CS and 94.6–5.4% for CCR) but was within the range for P4R5 to produce PEFA robustly at a high level (Fig. 2f).

Based on the determined optimal sugar concentration (Fig. 2a, b), the purified CS- and CCR-derived sugars and CC-xylose were condensed to 140 g/L and 120 g/L in glucose and xylose equivalence, respectively, for P4R5 fermentation. As shown in Fig. 4b, with CS- and CCR-derived sugars as the carbon source, the PEFA titers (24.1 ± 0.9 and 23.9 ± 0.6 g/L, respectively) and SCO contents (39.4% and 39.7%, respectively) were similar to that using pure glucose (Fig. 2a). The cell growth and lipid yields of P4R5 were also maintained at a high level. With CC-xylose as the carbon source, the cell growth and PEFA production were similar to that using pure xylose. The SCO content (29.2%) was even higher than the fermentation using 120 g/L pure xylose (26.0%) (Fig. 2b). This result indicated that the purified hydrolysates derived from non-food corn biomass could replace the grain-derived pure monosaccharides for P4R5 fermentation.

The P4R5 fermentation was further carried out in a 10-L fermenter using CCR-derived sugar as the carbon source. As shown in Fig. 4c, within 7-day cultivation, the final PEFA titer, cell biomass, and SCO content reached 43.8 ± 0.9 g/L, 41.9 ± 1.1 g/L, and 38.8 ± 1.8%, respectively. The simultaneous utilization of glucose and xylose was confirmed according to the HPLC analysis of the residual reducing sugar in the fermenter (Additional file 2: Fig S2a). The PEFA productivity was as high as 6.26 g/L/day. Such PEFA production performance was at a higher level than previous reports using pure glucose as the carbon source [25, 26]. Taken together, by combining the CBS process and P4R5 fermentation, we constructed a complete bioconversion process from non-food corn plant wastes to high-value-added PEFA and SCO.

### Semi-continuous PEFA production

**Fig. 5:**
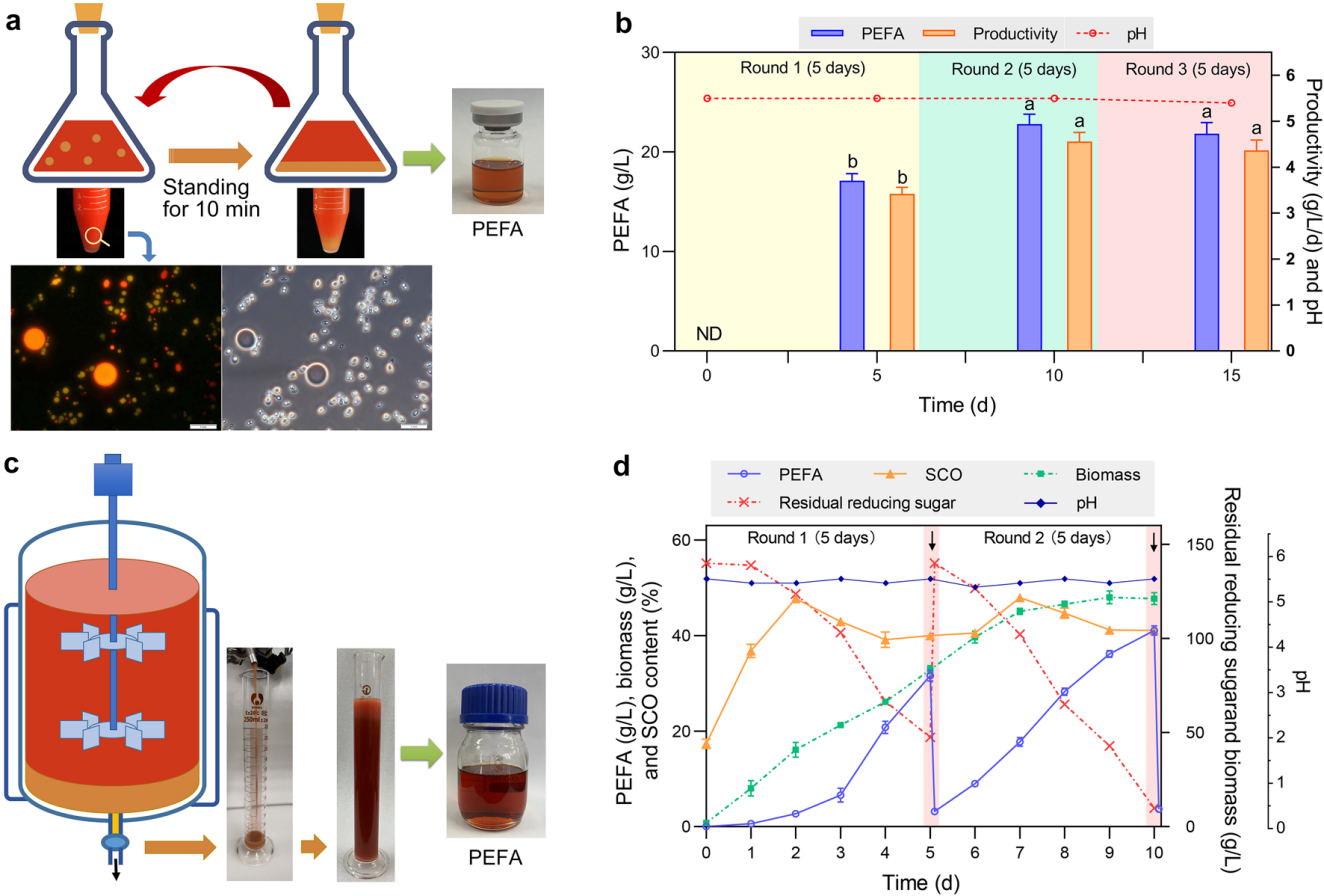
Semi-continuous fermentation of P4R5 with CCR-derived sugar as the carbon source. **a**, **c** Schematic representation of the semi-continuous fermentation of P4R5 for PEFA production in flasks and fermenter. **b** Three-round fermentation in 250-mL flasks. Each round of fermentation lasted for 5 days. The PEFA titer, PEFA productivity, and pH during fermentation were determined at the end of each round. ^abc^Data with different superscripts differ (*p* < 0.05). **d** Two-round fermentation in a 10-L fermenter. Each round of fermentation lasted for 5 days. The time course of PEFA titer, biomass, SCO content, residual reducing sugar, and pH during fermentation were determined. The concentration of glucose and xylose were also determined by HPLC analysis (Additional file 2: Fig S2b). The arrows indicate the time points for product collection and carbon source supplementation. Three replicates were prepared for statistical analysis

The density of PEFA is higher than oil-filled yeast cells, providing the possibility of convenient product separation. As shown in Fig. 5a, after standing for less than 10 min, the hydrophobic PEFA droplets could rapidly settle, forming a lower liquid layer separated from the culture. The yeast cells in the upper layer could be used for next-round cultivation by supplementing nutrients. The hydrophobic liquid in the lower layer could be directly used for PEFA extraction without centrifugation.

We first mimicked the semi-continuous process in 250-mL flasks with condensed CCR-derived sugar as the carbon source. According to our previous study, P4R5 requires about 7 days to reach maximal PEFA production [22]. Thus, the 1st round of fermentation lasted for 7 days. After standing for 10 min, 40-mL cell culture in the upper layer was transferred to 10 mL of fresh medium to initiate the 2nd and subsequently the 3rd round of fermentation. However, although the PEFA productivity increased, we detected lower PEFA production and cell growth in subsequent fermentations compared with the first round (Additional file 2: Fig S3). After 7-day cultivation, the cell growth was already in the stationary phase with less cell viability [22], which might lead to reduced production in the following rounds of fermentation. Therefore, the fermentation time was reduced to 5 days for each round.

In comparison with the 7-day fermentation in flasks, lower PEFA production was observed for the 1st round of fermentation due to the shortened cultivation time, but the subsequent PEFA production performance was kept at a high level similar to the batch fermentation. The PEFA titer was 22.8 ± 1.1 and 21.8 ± 1.3 g/L for the 2nd and 3rd rounds of fermentation, respectively (Fig. 5b). The PEFA productivity was even stimulated from 3.42 to 4.56 g/L/day and maintained at 4.36 g/L/day for the 2nd and 3rd round of fermentation, respectively. The enhanced productivity would help to reduce fermentation time and process costs. It is noteworthy that only 1.88 g/L of residual PEFA was detected in the upper layer of the culture after the 3rd separation, indicating that the separation efficiency of PEFA from the culture reached 92.0%. This result demonstrated that such semi-continuous fermentation and separation strategy was suitable for PEFA production by P4R5.

We further achieved the semi-continuous PEFA fermentation using a 10-L fermenter, which contained a sampling port at the bottom of the fermenting tank to facilitate the timely collection of produced PEFA droplets (Fig. 5c). Each round of fermentation was carried out for 5 days. Instead of transferring the upper-layer cells, concentrated CCR-derived sugar was supplemented into the fermenter after PEFA collection to initiate the next round of fermentation to simplify the operating process and reduce contamination risk. As shown in Fig. 5d, at the end of 1st round of fermentation, the PEFA titer, cell biomass, SCO content, and PEFA productivity reached 31.6 ± 1.1 g/L, 32.8 ± 0.9 g/L, 40.0 ± 0.9%, and 6.32 g/L/day, respectively. While at the end of 2nd round of fermentation, all the PEFA titer, cell biomass, SCO content, and PEFA productivity increased to 41.1 ± 0.9 g/L, 47.7 ± 1.2 g/L, 41.1 ± 0.8%, and 8.22 g/L/day, respectively.

At the end of the 1st round of fermentation, we observed 47.5 g/L of residual sugar and the sugar consumption rate significantly decreased on the fifth day. This might be caused by the slight inhibition effect of concentrated CBS sugars on cell growth. In contrast, over 93.0% of the supplemented sugar was utilized at the end of 2nd round of fermentation, and no significant delay in sugar consumption or PEFA production was observed. The better fermentation performance of the 2nd round of fermentation might benefit from the fast cell adaptation to the concentrated sugar. After PEFA collection at the end of each round of fermentation, less than 3.6 g/L of PEFA was detected in the culture, indicating a high separation efficiency of over 90%. During the semi-continuous fermentation, the pH value was stable at about 5.5 without any adjustment. All these results suggested the robustness of PEFA production by P4R5 through the semi-continuous fermentation and separation strategy.

## Discussion

Despite LCB material being known as a valuable and promising carbon source with sustainability [36], relatively few studies were devoted to the production of PEFA from non-food LCB due to the low cost-effectivity of lignocellulose hydrolysis and lack of robust fermenting strains [2, 8]. Here, by coupling whole-cell-based lignocellulose saccharification and yeast fermentation, we developed an integrated process to convert corn plant wastes, mainly corn stover and corncob residue, to PEFA and SCO (Fig. 1) and obtained the best glycolipid production performance for *Rhodotorula* species according to our best knowledge.

CBS is a recently developed lignocellulose saccharification technology using cellulosome-producing bacterial cells as whole-cell biocatalysts [5]. By introducing heterologous secretory β-glucosidase (BGL) proteins to release the cellobiose inhibition to the cellulosome system [37, 38], several biocatalysts have been developed with increasing saccharification efficiency [6, 7, 39]. It was reported that the substrate-to-sugar conversion ratio of the engineered *C.* *thermocellum* ∆*pyrF*::p2638-BGL could be over 75% with alkali-treated CCR as the substrate [39]. In this study, we used both CS and CCR, representing the main non-food waste of the corn plant, as the substrate for CBS and obtained the substrate-to-sugar conversion ratios of 81.2% and 76.3%, respectively. This result indicated the effective lignocellulose saccharification via the CBS process.

*R.* *paludigena* P4R5 was previously isolated from deep-sea sediment samples and could simultaneously produce intracellular SCO and extracellular PEFA at high levels with glucose as the sole carbon source [22]. Because glucose accounts for the majority of CBS-derived sugars [7], it is possible to integrate CBS with P4R5 fermentation for converting lignocellulosic corn plant wastes to PEFA and SCO. Nevertheless, the efficient utilization of CBS hydrolysates containing both C5 and C6 sugars by P4R5 must be confirmed. Our findings indicated that P4R5 could utilize various C5 and C6 monosaccharides from CBS hydrolysates, including glucose, xylose, and arabinose, for PEFA and SCO production (Fig. 2).

P4R5 could use mixed sugars with glucose-to-xylose ratios from 100%:0 to 75:25% robustly. It is noteworthy that the PEFA titers and yields and SCO content could be significantly influenced by further increasing xylose concentration (Fig. 2f). Similar to most yeasts [40], P4R5 assimilates xylose through xylose isomerization via xylitol, xylulose phosphorylation, and subsequent pentose phosphate pathway (Fig. 3c). The intermediate product glyceraldehyde-3-phosphate is further metabolized for PEFA and SCO synthesis through glycolysis. In addition, xylose reductase and xylitol dehydrogenase involved in the xylose isomerization usually prefer different cofactors [40], resulting in redox imbalance. Therefore, compared with the PEFA and SCO production from glucose, xylose metabolism is less efficient. However, according to our previous study, the glucan-to-xylan ratio in pretreated LCB biomass was usually higher than 75%:25%, and for CBS, the glucan saccharification efficiency could be similar to or higher than that of xylan [6, 39]. Indeed, we determined that glucan accounted for up to 79% and 92% of the polysaccharides in alkali-pretreated CS and CCR, respectively (Fig. 1). Therefore, the xylan-derived xylose in the LCB hydrolysates would not significantly influence P4R5 fermentation and PEFA production. Besides, it is noteworthy that PEFA produced by P4R5 from different monosaccharides showed similar surfactant properties and hydroxyl fatty acid profiles (Table 1; Fig. 3a). Taken together, P4R5 is a promising candidate for utilizing sugar-rich hydrolysates derived from non-food LCB biomass to produce high-value-added bioproducts.

In our previous study, the sugar-rich CBS hydrolysate has been employed for yeast fermentation to produce pullulan, but high loading of activated carbon (3%, w/v) was required for hydrolysate purification [12], leading to high costs. We reduced the dosage of activated carbon by 90% and elevated the reaction temperature to 90 °C to accelerate the adsorption process. Although the purified hydrolysate could be directly used for P4R5 fermentation, ion-exchange columns were further used to remove excess ammonia, considering the necessity of high initial C/N ratios to boost PEFA synthesis [22]. We compared the P4R5 fermentations with CBS hydrolysates or pure glucose as the carbon source and determined similar cell growth and PEFA and SCO production. The PEFA titers of P4R5 grown on CS- and CCR-derived sugars were significantly higher than those of the previously reported *Rhodotorula* strains, including *R.* *babjevae* Y-SL7 (3.3 g/L), *R.* *babjevae* UCDFST 04–830 (8.5 g/L), *R.* *paludigena* UCDFST 81–492 (11.7 g/L), and *R*. aff. *paludigena* UCDFST 81–84 (12.4 g/L), fermenting in flasks using glucose as the carbon source [25, 26]. This indicated that it is feasible to replace grain-derived sugars with CBS hydrolysates derived from corn plant wastes for PEFA and SCO production by P4R5.

As reviewed by Garay et al. [26], several successful attempts have been made for the simultaneous production of PEFA and SCO using *Rhodotorula* strains through batch fermentation. However, all reported processes utilized glucose or sucrose as the carbon resources, and PEFA and SCO titers were up to 31.2 and 8.8 g/L, respectively. We also performed batch fermentation of P4R5 using CCR-derived sugar in a 10-L bioreactor and simultaneously obtained 43.8 ± 0.9 g/L of PEFA and 16.3 ± 0.4 g/L of SCO after 7-day cultivation. The productivity was 6.26 and 2.33 g/L/day, respectively (Fig. 4c). Therefore, by using lignocellulose-derived sugar as the carbon source, P4R5 exhibited outstanding capability of PEFA and SCO synthesis and robust substrate utilization.

In comparison with traditional batch fermentation, continuous fermentation offers important advantages of higher productivity, improved product consistency, and reduced production cost [41]. Meantime, continuous fermentation requires specific separating technology or instrument for in-time product removal to avoid feedback inhibition. Because PEFA could settle rapidly after short-term standing, and no complex separation process was required, we determined to develop a semi-continuous process using CCR-derived hydrolysate as the carbon source (Fig. 5). During the semi-continuous fermentation, produced PEFA was easily collected from the sampling port at the bottom of the fermenting tank. The rest of the culture was directly used for the next round of fermentation by supplementing carbon sources, while no further cell inoculation is required. The results showed that the PEFA productivity was enhanced up to 8.22 g/L/day, which was the highest level to our best knowledge.

Compared with sophorolipid production which shows an average product separation efficiency ranging from 65.7% to 74.3% [42], over 90% of the produced PEFA could be separated from culture through short-time standing. Such high separation efficiency would benefit from the high-density and easy-to-settle properties of PEFA. The special fermentation ability of P4R5 could also contribute to the high separation efficiency of PEFA because, unlike sophorolipid-producing strains which use hydrophobic oils as the carbon source for fermentation [26], P4R5 can synthesize PEFA directly from water-soluble sugars to avoid the mixing of produced glycolipids with substrate oils.

During batch fermentation, we observed that the SCO concentration gradually decreased from about 50% to 40% after 48-h cultivation. When the SCO titer was maintained at about 40%, the PEFA production was enhanced and the PEFA concentration increased continuously (Fig. 4c). This phenomenon was also observed in our previous study [22]. For semi-continuous fermentation, we observed a repeated “increase–decrease–maintain” pattern for SCO content in the system for every round of fermentation. This indicated that the intracellular SCO could be produced as a transit to synthesize extracellular hydroxy fatty acids of PEFA. The balanced conversion of SCO to PEFA in the strain might be regulated based on the intracellular SCO content. The conversion might be catalyzed by an unidentified CYP52 cytochrome P450 [26].

In addition to being promising biosurfactants, PEFA produced by P4R5 from different carbon sources could also be used to prepare self-assembling nano-micelles (Table 1). Interestingly, P4R5 showed a unique capability of producing high-grade PEFA with a single arabitol or mannitol head, rather than mixed molecules, under a particular carbon resource condition (Fig. 3b). The regulation of sugar metabolism pathways responding to supplemented carbon sources was also investigated by transcriptional analysis (Fig. 3c). In contrast, as reviewed by Garay et al. [26], all known PEFA-producing strains only secrete mixtures of PEFA containing both d-mannitol and d-arabitol head groups, low amounts of xylitol were also detected in one study. It is reported that d-mannitol can act on the β3-adrenergic receptor of brown adipocytes to improve β-oxidation and energy expenditure [43], indicating that the single-polyol-head feature of PEFA could endow the prepared nano-micelles with unique targeting functions as smart drug-delivery nanocarriers. Thus, the capability of P4R5 to produce single-polyol-head PEFA would further extend its application in the medical field.

## Conclusions

At present, bioconversion of cheap agricultural wastes to high-value-added products is preferred for economic reasons. In this study, we established a complete bioconversion process to produce high-value-added glycolipids and lipids from non-food corn plant wastes (Fig. 1). Through the CBS process, both CS and CCR were effectively converted to sugar-rich hydrolysates. Then, the deep-sea yeast strain P4R5 utilized various CBS hydrolysates to produce extracellular PEFA and intracellular SCO simultaneously and robustly. Not only that, we provide a semi-continuous process for PEFA production from LCB with the highest titer, productivity, and separation efficiency hitherto known. Therefore, this work paves a promising way for the high-value-added utilization of agricultural wastes and provides a reference for PEFA production from non-food carbon sources.

## Methods

### Strains, media, and growth conditions

*R.* *paludigena* P4R5 was isolated from deep-sea sediment samples in the South Pacific [22], and was maintained in YPD medium at 28 °C. The PEFA production medium was composed of 140.0 g/L carbon sources, 1.0 g/L yeast extract, 1.0 g/L peptone, 7.0 g/L KH_2_PO4, 2.5 g/L Na_2_HPO_4_, and 1.5 g/L MgSO_4_•7H_2_O. *C.* *thermocellum* strain ∆*pyrF*::P_2638_-BGL [39] was cultivated anaerobically at 60 °C in GS-2 medium (1.5 g/L KH_2_PO_4_, 3.8 g/L K_2_HPO_4_·3H_2_O, 2.1 g/L urea, 1.0 g/L MgCl_2_·6H_2_O, 150 mg/L CaCl_2_·2H_2_O, 1.25 mg/L FeSO_4_·6H_2_O, 1.0 g/L cysteine–HCl, 10 g/L MOPS-Na, 6.0 g/L yeast extract, 3.0 g/L trisodium citrate·2H_2_O, 0.1 mg/L resazurin, pH 7.4) [44] with 5 g/L Avicel (PH-101, Sigma-Aldrich LLC.) as the carbon source.

### Genome sequencing, assembly, and annotation of P4R5

The genomic DNA of P4R5 was extracted and purified using the TIANamp yeast DNA kit (TIANGEN, Beijing, China). Sequencing libraries were generated using NEBNext^®^ Ultra™ DNA Library Prep Kit for Illumina (NEB, USA) following the manufacturer’s protocol. The whole genome of P4R5 was sequenced using Illumina NovaSeq PE150 and assembled with SOAP de novo software at the Beijing Novogene Bioinformatics Technology Co., Ltd. The coding genes were predicted using the Augustus 2.7 program, and the gene function was predicted based on the Non-Redundant Protein Database [45] and the Kyoto Encyclopedia of Genes and Genomes database. The genome information of P4R5 has been deposited to GenBank under the accession number ID: JAHERV000000000.

### RT-qPCR

RNA samples were isolated from P4R5 cells grown in the PEFA production medium at 28 °C for 72 h with different monosaccharides of d-glucose, d-xylose, d-fructose, d-arabinose, or l-arabinose as the carbon source. Purification of total RNA, quantification of synthesized cDNAs by qPCR (Additional file 3: Table S3), and data analysis were conducted essentially as described [46].

### Preparation of sugar-rich hydrolysates from non-food corn plant wastes through consolidated bio-saccharification

The corn plant biomass materials, including corncob (CC) and corn stover (CS), were collected from the countryside of Qingdao, China, and were air dried. Corncob was then pretreated by sulfuric acid-catalyzed hydrolysis to produce corncob-derived xylose (CC-xylose) and corncob residue (CCR) (Jinan Shengquan Group Shareholding Co. LTD). CCR and CS (cut into about 3 cm pieces) were pretreated using 10% (w/w) and 20% (w/w) NaOH for 60 min at 90 ºC and 140 ºC, respectively, with a liquid–solid ratio of 10. All solid materials were used for subsequent saccharification after washing to neutral with tap water. The main chemical composition of CS and CCR was determined based on the National Renewable Energy Laboratory analytical procedure [35].

The CBS process was performed using 40 g/L alkali-pretreated CS or CCR as the substrate to prepare the lignocellulosic hydrolysates as previously reported [6]. The strain *C.* *thermocellum* ∆*pyrF*::p2638-BGL [39] was initially cultivated with 5 g/L Avicel as the sole carbon source to the exponential stage and was inoculated into GS-2 medium containing alkali-pretreated CS or CCR to initiate the saccharification process. The obtained sugar-rich CBS hydrolysates derived from CCR and CS were first treated with 0.3% (w/v) activated carbon in a water bath at 90 °C shaking for 1 h based on a modified method [12] and were further purified using ion-exchange columns (Tulsion^®^ T-42H and Tulsimer^®^ A-23) to remove excess ammonia. The purified hydrolysates and CC-xylose were analyzed by the DNS method and high-performance liquid chromatography (HPLC) as previously described [7]. For batch and semi-continuous yeast fermentation, the purified hydrolysates were condensed to 140 g/L through reduced pressure distillation or spray-dried into powder.

### Yeast fermentation for PEFA production in flasks

P4R5 fermentation was carried out aerobically at 28 °C, 180 rpm for 7 days in 250-mL shake flasks containing 50 mL of PEFA production medium. Various monosaccharides, including d-glucose, d-xylose, d-fructose, d-arabinose, and l-arabinose, were used as carbon sources. The sugar concentration changed from 80 to 160 g/L when needed. When a mixture of xylose and glucose was used as the carbon source, the total sugar concentration was 140 g/L with varied glucose-to-xylose ratios (100%:0, 75%:25%, 50%:50%, 25%:75%, 0:100%). For fermenting with purified sugar-rich CBS hydrolysates as the nutrient, 120 g/L CC-xylose or 140 g/L CCR- or CS-derived sugar was supplemented.

To mimic semi-continuous fermentation in flasks, the first round of cultivation was carried out as described above using 140 g/L CCR-derived sugar as the carbon source and the cultivation lasted for 5 to 7 days. The culture was then transferred into sterile centrifuge tubes and stood for 10 min to separate the upper hydrophilic layer (mainly cell culture) and lower hydrophobic layer (mainly produced PEFA). Afterward, 40 mL of the upper-layer liquid was poured into 10 mL of fresh medium containing CCR-derived sugar for the second round of cultivation. The lower-layer liquid was used for PEFA extraction. The third round of cultivation was performed similarly. The sugar concentration was maintained at 140 g/L at the beginning of each round of cultivation. The PEFA titer, SCO content, and cell biomass were determined at the end of the fermentation following the methods below. To evaluate the performance of the standing separation process, PEFA separation efficiency was calculated by dividing the PEFA concentration difference by the initial PEFA concentration in the culture before separation [42].

### Batch and semi-continuous fermentation for PEFA production in a 10-L fermenter

Both batch and semi-continuous fermentation of P4R5 were performed in a 10-L fermenter using 140 g/L CCR-derived sugar as the carbon source. The sampling was performed every 12 or 24 h to determine the change in the cell biomass, PEFA and SCO production, and sugar consumption during fermentation. For semi-continuous fermentation, the extracellular PEFA droplets were collected every 5 days through a sampling port at the bottom of the fermenter. CCR-derived sugar powder was supplemented to start the next round of fermentation. The sugar concentration required for supplementation was calculated based on the residual sugar concentration and the remaining volume of the culture so that the initial sugar concentration (140 g/L) and initial total volume could be maintained for each round of fermentation.

### Microscopic visualization of PEFA and SCO

For microscopy analysis, 5.0 μL of the cell culture was mixed with 2.0 μL Nile red solution and stored at room temperature in darkness for 10 min. The mixture was then visualized using an Olympus U-LH100Hg fluorescence microscope in bright-field mode and fluorescence (530 nm excitation and 626 nm emission) with a magnification of 100 times.

### Extraction and analysis of extracellular PEFA, cell dry weight, and SCO

The preparation and analysis of extracellular PEFA, cell dry weight, and SCO were carried out according to our previous study [22]. Briefly, after fermentation, the PEFA in the lower layer was extracted with ethyl acetate from the culture and was quantitatively measured subsequently. The residue cell pellets were obtained from the upper layer of the culture by centrifugation, and freeze-dried to measure dry cell weight, [22]. The obtained dry cells were lysed in 6 mol/L HCl at 100 °C for 5 min, followed by extraction using chloroform–methanol solution (2:1, v/v). The SCO in the chloroform was vacuum dried for quantification.

The fatty acid composition of PEFA and SCO was analyzed through transmethylation and gas chromatography–mass spectrometry (GC–MS). The polyols of PEFA were prepared by alkaline hydrolysis using 2 M KOH and analyzed by HPLC using a Thermo UltiMate3000 system equipped with an evaporative light-scattering detector (SEDERE LT-ELSD Shodex 80) and an Asahipak NH2P-50 4E column. Xylitol, mannitol, and arabitol purchased from Sigma-Aldrich were used as the standards.

### Preparation and characterization of PEFA nano-micelles

The PEFA nano-micelles were prepared and characterized as previously reported [47]. Briefly, 1 mg/mL of PEFA dissolved in deionized water was subjected to ultrasonic treatment for 15 min (work for 2 s, pause for 2 s), and was diluted to a series of concentrations from 0.5 to 100 mg/L. The fluorescence probe technique based on the pyrene fluorescence assay [48] was used to determine CMC values of PEFA by plotting the ratio of *I*_*373*_/*I*_*383*_ vs. Log C (mg/L). The particle size, polymer dispersity index (PDI), and zeta potential of the prepared micelles were measured using Zetasizer Nano ZS90 (Malvern Instruments, UK).

### Statistical analysis

The statistical analysis was performed based on three separate experiments using the GraphPad Prism software. Means were compared and analyzed using one-way analysis of variance (ANOVA) with post hoc multiple comparisons (LSD and Waller–Duncan tests). Different letters within a column indicate significant differences (*p* < 0.05).

## Supplementary Information

**Additional file 1: Table S1. **Genome assembly and annotation statistics for the yeast strain P4R5.** Table S2** Relative expression level of the key genes involved in polyol synthesis under different carbon source conditions.

**Additional file 2:** **Fig S1**. HPLC analysis of the sulfuric acid hydrolysates of pretreated CS and CCR. The peaks referring to glucose, xylose, and arabinose were indicated. **Fig S2** The time courses of residual sugars during batch fermentation (a) and semi-continuous fermentation (b) of P4R5 as shown in Figs. 4c and 5d, respectively. The reducing sugar was determined by the DNS method. The glucose and xylose were analyzed by HPLC. Three replicates were prepared for statistical analysis. **Fig S3** Three-round fermentation of P4R5 with CCR-derived sugar as the carbon source in 250-mL flasks. The first round of fermentation lasted for 7 days and the second and third rounds of fermentation lasted for 5 days. The PEFA titer, PEFA productivity, and pH during fermentation were determined at the end of each round of fermentation.

**Additional file 3: Table S3.** qPCR primers for the key genes involved in polyol synthesis in P4R5.

## Data Availability

All data generated or analyzed during this study are included in this published article and its Additional files.

## Abbreviations

BGL: Beta-glucosidase
CBP: Consolidated bioprocessing
CBS: Consolidated bio-saccharification
CCR: Corncob residual
CMC: Critical micelle concentration
CS: Corn stover
DNS: 3,5-Dinitrosalicylic acid
GC–MS: Gas chromatography-mass spectrometry
HPLC: High-performance liquid chromatography
LCB: Lignocellulosic biomass
PDI: Polymer dispersity index
PEFA: Polyol esters of fatty acid
SCO: Single-cell oil
SSF: Simultaneous saccharification and fermentation
