# Supplementary material for: Bioconversion of non-food corn biomass to polyol esters of fatty acid and single-cell oils
Source: Biotechnol Biofuels Bioprod. 2023 Jan 17;16:9. doi: 10.1186/s13068-023-02260-z (PMC9844004; doi:10.1186/s13068-023-02260-z)
Supplement: Supplementary file 1 — Additional file 1: Table S1. Genome assembly and annotation statistics for the yeast strain P4R5. Table S2 Relative expression level of the key genes involved in polyol synthesis under different carbon source conditions. [file 13068_2023_2260_MOESM1_ESM.docx]

Table S1 Genome assembly and annotation statistics for the yeast strain P4R5

| General features | The yeast strain P4R5 |
| --- | --- |
| Length (bp) | 20,967,896 |
| Scaffolds | 277 |
| GC content (%) | 64.26 |
| Protein-coding gene number | 6,867 |
| Coding region of genome (%) | 52.48 |
| Total number of predicted ORFs | 6,867 |

Table S2 Relative expression level of the key genes involved in polyol synthesis under different carbon source conditions

| Carbon source | Gene name | Encoding product | Expression level (%)^a^ |
| --- | --- | --- | --- |
| Glucose | *ACTIN* | β-Actin | 100 ± 2.25 |
|  | *HXK* | Hexokinase | 41.40 ± 1.86 |
|  | *GLK* | Glucokinase | 23.94 ± 2.17 |
|  | *PGI1* | Glucose-6-phosphate isomerase | 49.55 ± 1.39 |
|  | *MPDH* | Mannitol-1-phosphate 5-dehydrogenase | 9.69 ± 0.72 |
|  | *M1PASE* | Mannitol-1-phosphatase | 40.90 ± 0.49 |
|  | *G6PD* | Glucose-6-phosphate 1-dehydrogenase | 108.49 ± 4.55 |
|  | *PGLS* | 6-Phosphogluconolactonase | 40.43 ± 1.05 |
|  | *PGD* | 6-Phosphogluconate dehydrogenase | 24.12 ± 0.61 |
|  | *RPE1* | Ribulose-phosphate 3-epimerase | 5.53 ± 0.05 |
|  | *TKL1* | Transketolase | 208.55 ± 5.40 |
|  | *XKS1* | Xylulose kinase | 0.84 ± 0.04 |
|  | *ARD1* | D-arabinitol dehydrogenase | 0.04 ± 0.00 |
|  | *RBTK* | D-ribulokinase | 13.74 ± 0.29 |
|  | *ARDH* | D-arabinitol 2-dehydrogenase | 13.26 ± 0.89 |
| Fructose | *ACTIN* | β-Actin | 100 ± 1.62 |
|  | *MtDH* | Mannitol 2-dehydrogenase | 171.95 ± 2.19 |
|  | *M1PASE* | Mannitol-1-phosphatase | 47.36 ± 2.56 |
|  | *HXK* | Hexokinase | 43.30 ± 2.20 |
|  | *MPDH* | Mannitol-1-phosphate 5-dehydrogenase | 10.65 ± 1.19 |
|  | *ARD1* | D-arabinitol dehydrogenase | 0.02 ± 0.01 |
|  | *ARDH* | D-arabinitol 2-dehydrogenase | 0.01 ± 0.00 |
| Xylose | *ACTIN* | β-Actin | 100 ± 2.58 |
|  | *ARD1* | D-arabinitol dehydrogenase | 14.00 ± 0.69 |
|  | *XKS1* | Xylulose kinase | 24.83 ± 1.52 |
|  | *RPE1* | Ribulose-phosphate 3-epimerase | 10.79 ± 1.31 |
|  | *RBTK* | D-ribulokinase | 16.29 ± 0.81 |
|  | *ARDH* | D-arabinitol 2-dehydrogenase | 12.68 ± 2.97 |
|  | *MtDH* | Mannitol 2-dehydrogenase | 57.05 ± 1.19 |
|  | *M1PASE* | Mannitol-1-phosphatase | 0.04 ± 0.01 |
|  | *GRE3* | Aldehyde reductase | 15.15 ± 0.45 |
|  | *XYL3* | D-xylose reductase | 11.89 ± 0.63 |
|  | *XYL2* | D-xylose reductase | 15.44 ± 0.86 |
|  | *TKL1* | Transketolase | 53.47 ± 0.98 |
|  | *MPDH* | Mannitol-1-phosphate 5-dehydrogenase | 12.13 ± 0.96 |
|  | *LAD1* | L-arabinitol 4-dehydrogenase | 0.08 ± 0.02 |
|  | *XYL1* | L-xylulose reductase | 9.91 ± 0.53 |
|  | *XKIA* | L-xylulokinase | 2.39 ± 0.09 |
|  | *SGBU* | PRL-ribulose-5-phosphate 3-epimerase | 50.25 ± 1.53 |
|  | *RPE2* | Ribulose-phosphate 3-epimerase | 0.75 ± 0.02 |
| D-arabinose | *ACTIN* | β-Actin | 100 ± 3.76 |
|  | *FUCI* | D-arabinose isomerase | 5.79 ± 0.68 |
|  | *ARDH* | D-arabinitol 2-dehydrogenase | 13.23 ± 1.00 |
|  | *ARD1* | D-arabinitol dehydrogenase | 0.03 ± 0.01 |
|  | *M1PASE* | Mannitol-1-phosphatase | 0.02 ± 0.00 |
| L-arabinose | *ACTIN* | β-Actin | 100 ± 3.60 |
|  | *GRE3* | Aldehyde reductase | 19.52 ± 0.41 |
|  | *LAD1* | L-arabinitol 4-dehydrogenase | 139.76 ± 4.19 |
|  | *XYL1* | L-xylulose reductase | 9.17 ± 0.19 |
|  | *XYL2* | D-xylose reductase | 6.53 ± 0.52 |
|  | *ARD1* | D-arabinitol dehydrogenase | 10.09 ± 0.43 |
|  | *XKS1* | Xylulose kinase | 0.02 ± 0.01 |
|  | *RPE1* | Ribulose-phosphate 3-epimerase | 4.48 ± 0.01 |
|  | *RBTK* | D-ribulokinase | 19.57 ± 0.53 |
|  | *XKIA* | L-xylulokinase | 1.40 ± 0.11 |
|  | *SGBU* | L-ribulose-5-phosphate 3-epimerase | 19.68 ± 0.18 |
|  | *RPE2* | Ribulose-phosphate 3-epimerase | 5.64 ± 0.45 |
|  | *ARDH* | D-arabinitol 2-dehydrogenase | 78.73 ± 5.81 |
|  | *TKL1* | Transketolase | 52.11 ± 1.76 |
|  | *MPDH* | Mannitol-1-phosphate 5-dehydrogenase | 3.37 ± 1.09 |
|  | *M1PASE* | Mannitol-1-phosphatase | 8.80 ± 0.97 |

^a^ the transcriptional intensity of *β-ACTIN* in P4R5 grown on different sugars was determined as 100% to calculate the relative expression level of selected genes.
