# Supplementary figures and images for: Bioconversion of non-food corn biomass to polyol esters of fatty acid and single-cell oils
Source: Biotechnol Biofuels Bioprod. 2023 Jan 17;16:9. doi: 10.1186/s13068-023-02260-z (PMC9844004; doi:10.1186/s13068-023-02260-z)

Fig. S1


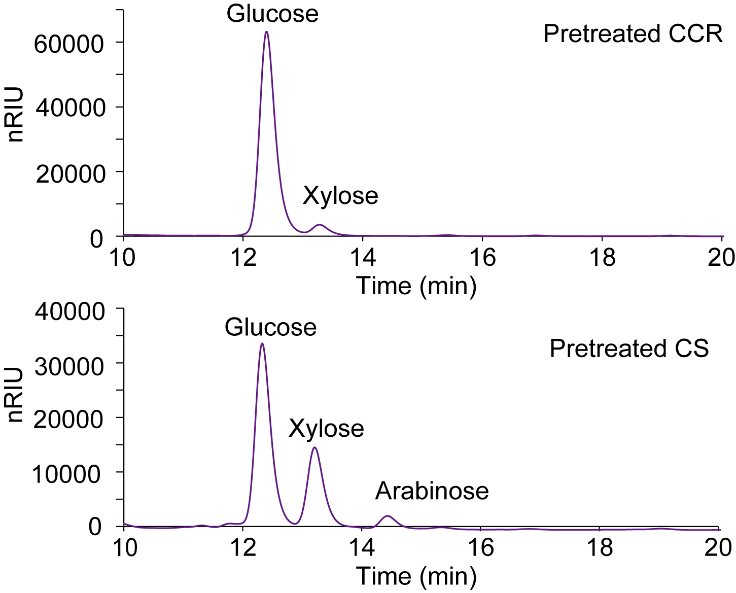


Fig. S2


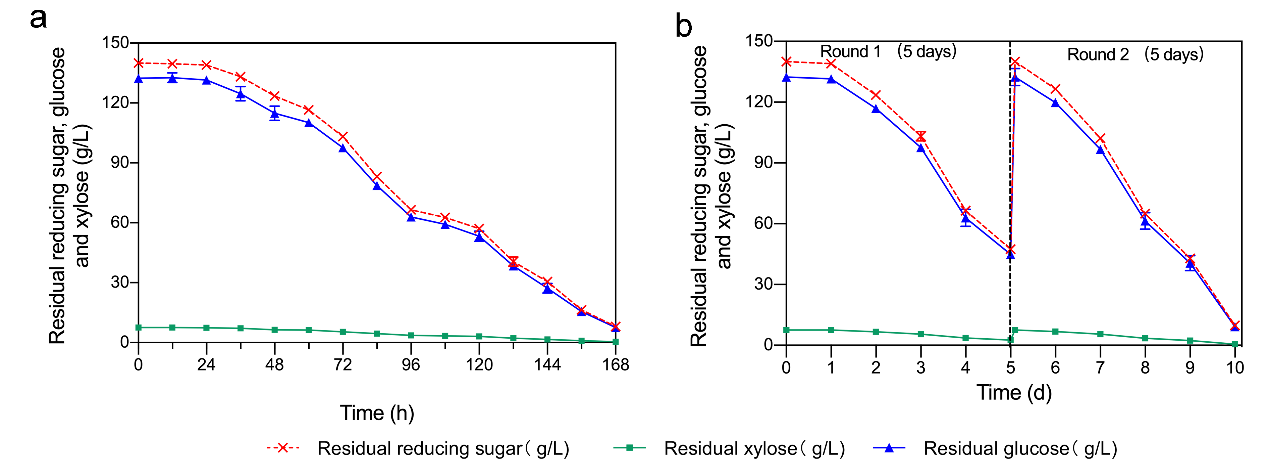


Fig. S3


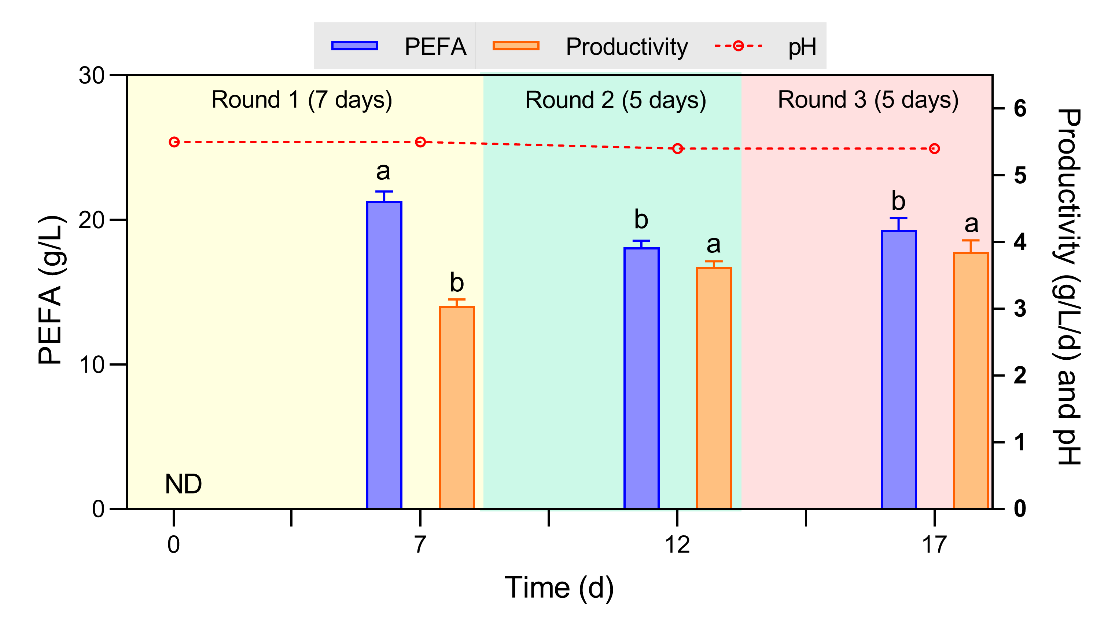

Supplement: Supplementary file 2 — Additional file 2: Fig S1. HPLC analysis of the sulfuric acid hydrolysates of pretreated CS and CCR. The peaks referring to glucose, xylose, and arabinose were indicated. Fig S2 The time courses of residual sugars during batch fermentation (a) and semi-continuous fermentation (b) of P4R5 as shown in Figs. 4c and 5d, respectively. The reducing sugar was determined by the DNS method. The glucose and xylose were analyzed by HPLC. Three replicates were prepared for statistical analysis. Fig S3 Three-round fermentation of P4R5 with CCR-derived sugar as the carbon source in 250-mL flasks. The first round of fermentation lasted for 7 days and the second and third rounds of fermentation lasted for 5 days. The PEFA titer, PEFA productivity, and pH during fermentation were determined at the end of each round of fermentation. [file 13068_2023_2260_MOESM2_ESM.docx]
